# Effect of Carotid Artery Morphological Variations on Cognitive Function

**DOI:** 10.1155/2018/7290431

**Published:** 2018-08-16

**Authors:** Lin Chen, Jialu Huang, Suxia Wang, Hong Ran, Lan Wen, Kangning Chen, Zhenhua Zhou

**Affiliations:** ^1^Department of Neurology, Southwest Hospital, Third Military Medical University, Chongqing 400038, China; ^2^Department of Pain, Southwest Hospital, Third Military Medical University, Chongqing 400038, China

## Abstract

**Background:**

Carotid artery morphological variations (CAMV) are common variations on medical imaging; the effects of CAMV on cognition were still unknown. This study is aimed at investigating whether carotid artery morphological variations (CAMV) cause cognitive impairment.

**Methods:**

Hospitalized patients from March 2017 to October 2017 who underwent digital subtract angiography (DSA) were divided into non-CAMV group, T-type group, K-type group, and C-type group according to their carotid artery morphology. Cognitive function in each group was evaluated with the Mini-Mental State Scale (MMSE), the Montreal Cognitive Assessment (MoCA), the Verbal Fluency Test (VFT), and the Digital Span Test (DST).

**Results:**

A total of 96 patients were included in the study (32 in non-CAMV group, 34 in T-type group, 30 in K-type group, and none in C-group). The positive rate of MMSE in the non-CAMV group, the T-type group, and the K-type group was 15.6%, 14.7%, and 20.0%, respectively, with no statistical difference in the three groups (*p* = 0.836). The positive rate of MoCA in the K-type group was significantly higher than that in the non-CAMV and the T-type groups (*p* < 0.001), but there was no significant difference between the non-CAMV group and the T-type group (*p* = 0.826). The VFT, DST forward score, and backward score in the K-type group were significantly lower than those in the non-CAMV and the T-type groups (*p* < 0.001).

**Conclusions:**

K-type CAMV may cause cognitive impairment, and MoCA is superior to MMSE in identifying mild cognitive impairment caused by CAMV.

## 1. Introduction

With the advent of the ageing of human society, the incidence of cognitive impairment especially vascular cognitive impairment has gradually increased and has caused widespread concern. Previous studies have found that chronic cerebral ischemia may result in cognitive impairment and become the main cause of vascular dementia [1].

Carotid artery morphological variations (CAMV) are common variations on medical imaging. It refers to the morphological characteristics of carotid artery that are caused by the extension of the carotid artery [2]. Previous studies have found that CAMV was associated with ischemic stroke and carotid artery dissection which may lead to ischemic changes [3–6]. However, the effects of CAMV on cognition were still unknown. In the present study, neuropsychological scales were used to evaluate the cognitive function of patients who were confirmed with CMAV with DSA and to further explore the effects of CAMV on cognitive function.

## 2. Methods

### 2.1. Study Subjects

We collected hospitalized patients who underwent digital subtract angiography (DSA) in our department from March 2017 to October 2017, and patients of CAMV were divided into three types: tortuosity (T), kinking (K), and coiling (C) according to the Weibel classification criteria [7]: (1) T-type: artery forming “S” or “C”-like tortuous with an angle of 90–165°; (2) K-type: arterial forming “<” or “>”-like flexion with an angle of 0–90°; (3) C-type: the arteries form an “O”-like curve with an angle of 360°. According to the DSA results, patients were divided into four groups: T-type group, K-type group, C-type group, and non-CAMV group. All patients signed consent form, and the study was approved by the Ethics Committee of Southwest Hospital of Third Military Medical University. Inclusion criteria were as follows: (1) age ≥ 18 years with no definite neurological disorders; (2) capable to complete neuropsychological examinations; and (3) patient and family members were well informed and signed a consent form. Exclusion criteria were as follows: (1) neurological diseases that may cause cognitive impairment, such as acute cerebrovascular disease, brain trauma, brain tumor, hydrocephalus, Parkinson's disease, multiple sclerosis, and central nervous system infections; (2) cerebral artery stenosis or occlusion and cerebral vascular malformation with DSA examination; (3) history of mental illness, dementia, and taking medications that may affect cognitive function; (4) years of education ≤ 1 year; (5) severe visual impairment, audition dysfunctions, and aphasia; and (6) other causes that may induce cognitive impairment.

### 2.2. Neuropsychological Assessments

Patients completed neuropsychological assessments from 2 days to 1 week after the DSA examination. Examination scales were Mini-Mental State Scale (MMSE), Montreal Cognitive Assessment (MoCA), Verbal Fluency Test (VFT), and Digital Span Test (DST). The evaluations were independently completed by physicians who were blind to the study design with qualifications for neuropsychological assessment. (1) MMSE includes time-spatial orientation, immediate memory, calculation and attention, delayed recall, object naming, language repetition, reading comprehension, language understanding, language expression, and visual space perception. The total score is 30, abnormal < 27. (2) MoCA includes visuospatial and executive functions, naming, instant memory, calculations and attention, language retelling, abstraction, delayed recall, and temporal-spatial orientation. The total score is 30, normal ≥ 26, and add 1 point if years of education were less than 12 years. (3) DST presents a set of numbers. The subjects immediately repeat the sentences after listening. Memory span scores for forward and backward were recorded as the number of items in the longest series correctly recalled. (4) VFT requires the subjects to name the animal as much as possible within one minute.

### 2.3. DSA Assessment

Flat-panel digital subtraction imaging system was used (GE NOVA 4100, USA). The DSA data were independently analyzed by two experienced imaging experts who were blind to the study design.

### 2.4. Statistical Analysis

SPSS 23.0 software was used for statistical analysis. One-way ANOVA was used for continuous data of multiple groups, and multiple comparisons were performed with LSD *t*-test. The chi-squared test was used to compare multiple groups of counting data for multiple comparisons. Statistical significance was set at a *p* value < 0.05.

## 3. Results

### 3.1. Characteristics of Included Patients

**Figure 1:**
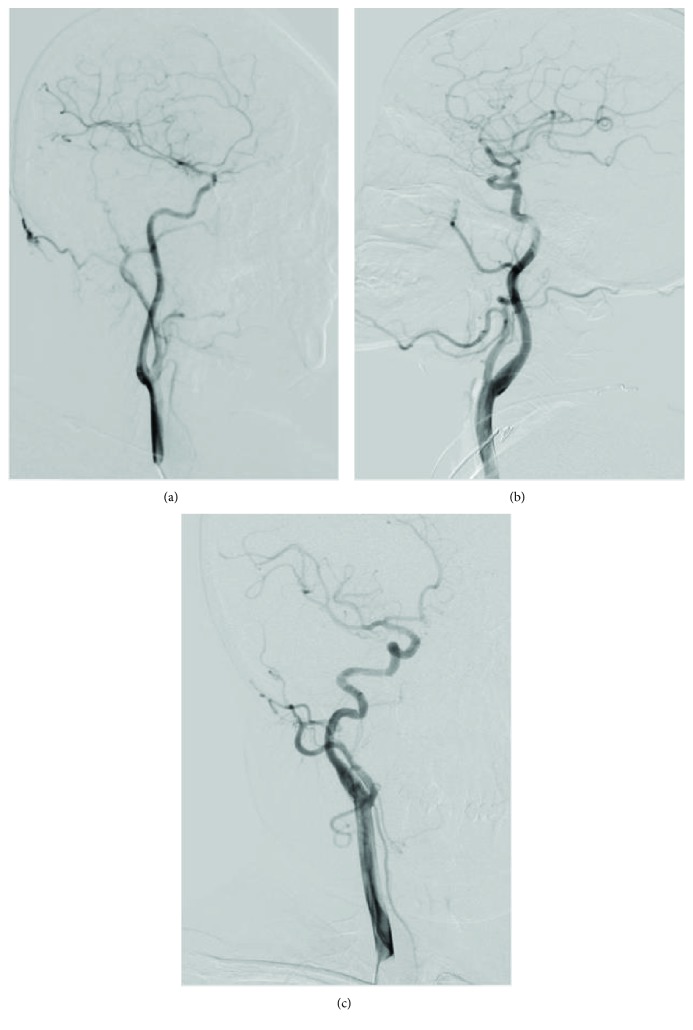
(a) Non-CAMV group. (b) T-type group. (c) K-type group.

A total of 96 patients were included in this study, including 54 males and 42 females. The mean age (65.8 ± 10.1) ranges from 45 to 86. There were 32 cases in the non-CAMV group, 34 cases in the T-type group, 30 cases in the K-type group, and none in the C-type group. Therefore, multiple comparisons were performed in the non-CAMV group, the T-type group, and the K-type group (Figure 1). There were no significant differences on sex ratio, age, years of education, smoking, drinking, diabetes, hyperlipidemia, and coronary heart disease (*p* > 0.05) (Table 1).

### 3.2. The Neuropsychological Examinations

**Figure 2:**
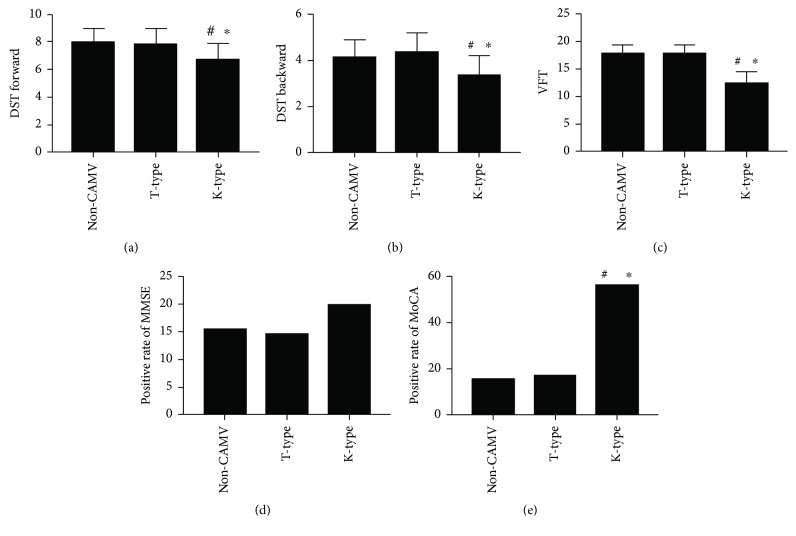
Multiple comparisons among the three groups on DST forward, DST backward, VFT, positive rate of MMSE, and positive rate of MoCA. ^#^Comparison with the non-CAMV group, *p* < 0.05. ^∗^Comparison with the T-type group, *p* < 0.05.

(1) MMSE: 5 of 32 patients (15.6%) in the non-CAMV group were abnormal; 5 of 34 patients (14.7%) in the T-type group were abnormal; 6 of 30 patients (20.0%) in the K-type group were abnormal. The positive rate of MMSE in each group was not statistically significant (*p* = 0.836). (2) MoCA: 5 of 32 patients (15.6%) in the non-CAMV group were abnormal; 6 of 34 patients (17.6%) in the T-type group were abnormal; 17 of 30 patients (56.7%) in the K-type group were abnormal. The positive rate of MoCA in the K-type group was significantly higher than that in the non-CAMV group and the T-type group (*p* < 0.001), but there was no statistical difference between the non-CAMV group and the T-type group (*p* = 0.826). (3) DST forward score: the non-CAMV group was 8.0 ± 1.0, the T-type group was 7.9 ± 1.1, and the K-type group was 6.8 ± 1.1. There was a significant difference between the K-type group and the non-CAMV group or the T-type group (*p* < 0.001), but there was no significant difference between the non-CAMV group and the T-type group (*p* = 0.734). (4) DST backward score: the non-CAMV group was 4.2 ± 0.7, the T-type group was 4.4 ± 0.8, and the K-type group was 3.4 ± 0.8. There was a significant difference between the K-type group and the non-CAMV or the T-type group (*p* < 0.001), but there was no significant difference between the non-CAMV group and the T-type group (*p* = 0.254). (5) VFT: the non-CAMV group was 17.9 ± 1.5, the T-type group was 18.0 ± 1.4, and the K-type group was 12.6 ± 1.9. There was a significant difference between the K-type group and the non-CAMV/T-type group (*p* < 0.001). There was no significant difference between the non-CAMV group and the T-type group (*p* = 0.815) (Figure 2).

### 3.3. Comparison of MMSE and MoCA on the Detection of Cognitive Impairment

MMSE detected 16 cases of cognitive impairment, accounting for 16.7% of the total patients; MoCA detected 28 cases accounting for 29.2% of the total patients. There were statistical differences between the two neuropsychological examinations (*p* = 0.039).

## 4. Discussion

Vascular dementia (VaD) is the only reversible dementia and the second most common dementia after Alzheimer's disease (AD). Bowler and Hachinski [8] proposed the concept of vascular cognitive impairment (VCI) for early detection and early intervention in 1995. To date, the concept of VIC has been expanded to a series of syndromes of mild to severe cognitive dysfunction resulting from cerebrovascular disease and its risk factors [9, 10]. Previous studies found that chronic cerebral ischemia is an important factor in the onset and development of vascular cognitive impairment [1]. There are many reasons for chronic cerebral ischemia, among which, large vessel stenosis is the most common. Wang et al. [11] studied and found that cerebral blood flow improved with revascularization after internal carotid artery stripping in patients with internal carotid artery stenosis > 65%. Yoshida et al. [12] achieved similar conclusions with a dynamic positron emission tomography method using 18F-2-deoxyglucose as a tracer of glucose metabolism to reflect the cerebral blood flow in patients with internal carotid artery stenosis. Collectively, those previous results suggested that large vascular stenosis can induce low perfusion cerebral of blood flow, leading to cognitive dysfunction.

CAMV is a common radiologic finding in the elderly population, and its incidence gradually increases with age. Distorted blood vessels influence flow dynamics and result in a series of clinical events. Wang et al. [13] used the anatomical parameters of the internal carotid artery as the prototype to establish an arterial distortion model. They simulated the blood flow dynamics and found that the distorting internal carotid artery could induce distal lower blood pressure. Derrick et al. [14] found that when the angle of carotid artery reaches 60°, the blood flow can be reduced by more than 40% and when the angle reaches 30°, the blood flow is reduced by more than 60%. Alexandrov [15] found that the reduced blood flow caused by the flexed carotid artery was comparable to the carotid stenosis with a stenosis of 75%. It can be concluded that the morphorgical abnormality of carotid arteries leads to chronic cerebral ischemia through the hemodynamics and could probably further damage cognitive function.

Our findings confirm this statement. Our study found that there was a significant difference between the K-type group and the non-CAMV group/T-type group on MoCA positive rate, VFT scores, and DST scores. However, no significance was observed in the T-type group compared to the non-CAMV group. The possible reason is that different degrees of vascular angulation have different effects on hemodynamics and may also have different effects on cognitive function. There have been few reports on the effects of carotid morphological variations on cognitive function. Zhou et al. [16] conducted follow-up of 1741 patients (over 50 years of age) who had completed CTA examination and diagnosed carotid and vertebral artery morphological changes. Severe carotid and vertebral artery morphological variations increased the incidence of Alzheimer's disease (AD).

In present study, the MoCA detected cognitive abnormalities in 28 cases of the three groups, while MMSE only detected 16 cases. Studies have shown that MoCA has good sensitivity and specificity for patients with mild cognitive impairment. Compared to MMSE, MoCA increased the weight of scores on executive function and attention and increased the sensitivity for early detection of mild cognitive impairment (MCI) patients with executive function and attention impairment. Meta-analysis by Ciesielska et al. [17] found that the detection rate of mild cognitive impairment in MoCA patients over 60 years old was better than MMSE; Trzepacz et al. [18] found meta-analysis that MoCA and MMSE had similar diagnostic value for dementia. However, in the diagnosis of mild cognitive impairment, the ceiling effect of MoCA is lower than that of MMSE. In addition, we observed a significant difference in DST and VFT scores between the K-type group and the non-CAMV group/T-type group. VFT is easy to operate and is widely used in the diagnosis and differential diagnosis of cognitive dysfunction [19]. It focuses on the function of the temporal lobe. DST which tests attention and memory is part of Wechsler Memory Scale and Wechsler Intelligence Scale. The results of this study suggested that there may be cognitive impairment in the K-type group, including attention and memory.

## 5. Limitation

There were also some limitations in this study. Firstly, we observed that CAMV may lead to cognitive impairment and this result may be due to chronic cerebral ischemia. However, this inference has not been verified by MR perfusion-weighted imaging (PWI) or computed tomography perfusion (CTP). We will further explore this in our future research. Secondly, the number of cases in this study is limited, and to elucidate the effect of CAMV on cognitive function requires a larger sample of patients.

## 6. Conclusions

Our study found that there was a significant difference between the K-type group and the non-CAMV group/T-type group on MoCA positive rate, VFT scores, and DST scores; no significance was observed in the T-type group compared to the non-CAMV group. K-type CAMV may cause cognitive impairment; the possible reason is that K-type CAMV can influence on hemodynamics. Furthermore, MoCA is superior to MMSE in identifying mild cognitive impairment caused by CAMV.

## Figures and Tables

**Table 1:** Demographic characteristics of the three groups.

	Non-CAMV	T-type	K-type	*p* value
Age, y	66.1 ± 10.0	65.9 ± 10.6	65.4 ± 10.0	0.968
Male, *n* (%)	18 (56.3)	19 (55.9)	17 (56.7)	0.998
Education, y	6.7 ± 3.6	7.4 ± 3.6	7.0 ± 3.5	0.685
Smoking, *n* (%)	10 (31.3)	12 (35.3)	12 (40.0)	0.772
Drinking, *n* (%)	12 (37.5)	13 (38.2)	12(40.0)	0.979
Diabetes mellitus, *n* (%)	11 (34.4)	14 (41.2)	12(40.0)	0.835
Hypertension, *n* (%)	17 (53.1)	18 (52.9)	15(50.0)	0.963
Hyperlipidemia, *n* (%)	16 (50.0)	14 (41.2)	15(50.0)	0.709
Coronary artery disease, *n* (%)	12 (37.5)	14 (41.2)	10(33.3)	0.811

## Data Availability

The data used to support the findings of this study are available from the corresponding author upon request.

## References

[1] Kim H. A., Miller A. A., Drummond G. R. (2012). Vascular cognitive impairment and Alzheimer’s disease: role of cerebral hypoperfusion and oxidative stress. *Naunyn-Schmiedeberg's Archives of Pharmacology*.

[2] Metz H., Murray-Leslie R. M., Bannister R. G., Bull J. W., Marshall J. (1961). Kinking of the internal carotid artery. *Lancet*.

[3] Vannix R. S., Joergenson E. J., Carter R. (1977). Kinking of the internal carotid artery. Clinical significance and surgical management. *American Journal of Surgery*.

[4] Saba L., Argiolas G. M., Sumer S. (2015). Association between internal carotid artery dissection and arterial tortuosity. *Neuroradiology*.

[5] Barbour P. J., Castaldo J. E., Rae-Grant A. D. (1994). Internal carotid artery redundancy is significantly associated with dissection. *Stroke*.

[6] Baracchini C., Farina F., Tonello S. (2013). Endothelial dysfunction in carotid elongation. *Journal of Neuroimaging*.

[7] Weibel J., Fields W. S. (1965). Tortuosity, coiling, and kinking of the internal carotid artery. I. Etiology and radiographic anatomy. *Neurology*.

[8] Bowler J. V., Hachinski V. (1995). Vascular cognitive impairment: a new approach to vascular dementia. *Baillière's Clinical Neurology*.

[9] Hachinski V., Iadecola C., Petersen R. C. (2006). National Institute of Neurological Disorders and Stroke-Canadian Stroke Network vascular cognitive impairment harmonization standards. *Stroke*.

[10] Gorelick P. B., Scuteri A., Black S. E. (2011). Vascular contributions to cognitive impairment and dementia: a statement for healthcare professionals from the American Heart Association/American Stroke Association. *Stroke*.

[11] Wang Q., Zhou M., Zhou Y., Ji J., Raithel D., Qiao T. (2015). Effects of carotid endarterectomy on cerebral reperfusion and cognitive function in patients with high grade carotid stenosis: a perfusion weighted magnetic resonance imaging study. *European Journal of Vascular and Endovascular Surgery*.

[12] Yoshida K., Ogasawara K., Saura H. (2015). Post-carotid endarterectomy changes in cerebral glucose metabolism on 18F-fluorodeoxyglucose positron emission tomography associated with postoperative improvement or impairment in cognitive function. *Journal of Neurosurgery*.

[13] Wang L. J., Wang D. M., Zhao F. (2008). Clinical study and numerical simulation of hemodynamics in the tortuosity of internal carotid artery. *Zhonghua Wai Ke Za Zhi*.

[14] Derrick J. R., Estess M., Williams D. (1965). Circulatory dynamics in kinking of the carotid artery. *Surgery*.

[15] Alexandrov A. V. (2007). The Spencer’s curve: clinical implications of a classic hemodynamic model. *Journal of Neuroimaging*.

[16] Zhou R., Liu D., Yu K. (2015). Carotid and vertebral arterial variations in Alzheimer’s disease. *Current Alzheimer Research*.

[17] Ciesielska N., Sokołowski R., Mazur E., Podhorecka M., Polak-Szabela A., Kędziora-Kornatowska K. (2016). Is the Montreal Cognitive Assessment (MoCA) test better suited than the Mini-Mental State Examination (MMSE) in mild cognitive impairment (MCI) detection among people aged over 60? Meta-analysis. *Psychiatria Polska*.

[18] Trzepacz P. T., Hochstetler H., Wang S., Walker B., Saykin A. J., for the Alzheimer’s Disease Neuroimaging Initiative (2015). Relationship between the Montreal Cognitive Assessment and Mini-mental State Examination for assessment of mild cognitive impairment in older adults. *BMC Geriatrics*.

[19] Henry J. D., Crawford J. R., Phillips L. H. (2004). Verbal fluency performance in dementia of the Alzheimer’s type: a meta-analysis. *Neuropsychologia*.

